# Postprandial Effects of Four Test Meals Containing Wholegrain Rye or Refined Wheat Foods on Circulating Incretins, Ghrelin, Glucose, and Inflammatory Markers

**DOI:** 10.1016/j.tjnut.2024.10.046

**Published:** 2024-11-06

**Authors:** Sebastian Åberg, Dominic-Luc Webb, Elise Nordin, Per M Hellström, Rikard Landberg

**Affiliations:** 1 Department of Life Sciences, Division of Food and Nutrition Science, Chalmers University of Technology, Gothenburg, Sweden; 2 Department of Medical Sciences, Gastroenterology and Hepatology, Uppsala University, Uppsala, Sweden

**Keywords:** wholegrain rye, obesity, overweight, continuous glucose, ghrelin, incretin, appetite regulation, postprandial glucose, postprandial incretin, clinical trial

## Abstract

**Background:**

High intake of whole grains has consistently been associated with reduced risk of obesity, coronary artery disease, and type 2 diabetes. Dietary interventions have shown beneficial metabolic effects of whole grains, but the metabolic response varies with different types of cereals.

**Objectives:**

We evaluate the metabolic effects of substituting refined wheat with wholegrain rye foods within a complex diet, examining the day-long postprandial response of glucose-dependent insulinotropic peptide (GIP), glucagon-like peptide-1 (GLP-1), ghrelin, glucose, and inflammatory biomarkers in individuals with overweight and obesity.

**Methods:**

Twenty-nine healthy adults with body mass index of 32 ± 9 kg/m^2^ were randomly assigned to 3 intervention days, separated by 1-wk washout. Participants adhered to a hypocaloric diet rich in wholegrain rye for 1 intervention and refined wheat for the second intervention and were randomly assigned to either diet for the third intervention with continuous blood sampling.

**Results:**

No differences in GIP, GLP-1, or ghrelin levels were found between the diets when measured throughout the whole intervention day. GIP total area under the curve after the rye-based lunch was 31% (*P* < 0.05) lower compared with the wheat-based lunch, and ghrelin concentrations were 29% (*P* < 0.05) lower after the rye-based dinner. Baseline Homeostatic Model Assessment for Insulin Resistance-adjusted model showed 61% (*P* = 0.015) lower whole-day GLP-1 and 40% (*P* = 0.03) lower GIP after the rye-based diet. Day-long glucose incremental area under the curve was 30% (*P* < 0.001) lower after the rye-based diet, and glycemic variability was measured as SD reduced (−0.13 mmol/L, *P* = 0.04). The rye-based diet compared with refined wheat induced higher glycoprotein N-acetylation A, as measured by z-scores (0.36, *P* = 0.014).

**Conclusions:**

Overall, no day-long differences in gut hormone levels were observed, but the wholegrain rye-based compared with refined wheat-based dinner showed lower postprandial ghrelin concentrations. The rye-based diet improved day-long glycemic control in individuals with overweight and obesity. Observations of diet-induced inflammation after whole-grain rye intake warrant further investigation.

**Trial registration number:**

This study was registered at Clinical Trials Registry of clinicaltrials.gov (NCT05004584): https://clinicaltrials.gov/study/NCT05004584?locStr=Gothenburg,%20Sweden&country=Sweden&state=V%C3%A4stra%20G%C3%B6taland%20County&city=Gothenburg&distance=50&term=appetite&aggFilters=status:com&rank=1.

## Introduction

Diets rich in whole grains have consistently been associated with reduced incidence of obesity, coronary artery disease, type 2 diabetes, colorectal cancer, and all-cause mortality [1]. Consequently, official dietary guidelines in many countries advocate for increased consumption of wholegrain foods. Acute meal studies and long-term dietary interventions have shown beneficial metabolic effects of wholegrain foods, including improvements in glycemia, blood lipid profiles, inflammation, and gut hormones. However, the metabolic responses differ between grains and there is large heterogeneity across studies. The appetite-regulating properties of foods may be of importance for development of overweight and obesity and its prevention [2,3]. Wholegrain foods have shown beneficial effects on subjective appetite control when compared with refined alternatives [4], particularly wholegrain rye [[5], [6], [7], [8]]. Wholegrain rye has the highest content of dietary fiber among all cereals and has consistently shown improved subjective appetite compared with isocaloric refined wheat products [4]. However, there is a lack of studies in obese and free-living individuals, where effects have appeared less pronounced [9]. Moreover, postprandial gut hormone response as objective markers of appetite has shown inconclusive results [5,7,10,11].

Commonly consumed refined cereals elicit a high glycemic response, and substitution with whole grain alternatives has emerged as a dietary strategy to improve glycemic control. Specifically for wholegrain rye, some intervention studies have shown improved acute meal responses, whereas others report no difference in glycemic response compared with refined alternatives [12].

The whole-day glycemic response or glycemic variability after diets rich in wholegrain rye has not yet been studied. Although, whole grains have shown potential to lower inflammation when part of the diet, especially in individuals with overweight or obesity [13], inflammatory biomarkers have rarely been studied during the postprandial period.

Novel inflammation markers glycoprotein N-acetylation (GlycA and GlycB) and supramolecular phospholipid composite peak (SPC) detected by nuclear magnetic resonance (NMR) spectroscopy can give important systemic metabolic information [14]. Elevated GlycA concentrations have been shown to predict the risk of type 2 diabetes mellitus, cardiovascular disease, and all-cause mortality [15], and both GlycA and GlycB have been suggested to better reflect inflammation than traditional inflammatory markers such as C-reactive protein (CRP) and IL-6 [16,17]. SPC mainly correlates with apolipoproteins levels [18]. Additionally, GlycA has recently been identified as a promising candidate biomarker for assessing diet-induced inflammatory response during the postprandial phase [19].

The aim of this study was to evaluate effects of replacing refined wheat cereals with wholegrain rye cereals within a complex diet on gut hormones that are relevant in reflecting postprandial appetite response: glucose-dependent insulinotropic peptide (GIP), glucagon-like peptide-1 (GLP-1), peptide tyrosine tyrosine (PYY), ghrelin and blood glucose control in individuals with overweight and obesity. In addition, we aimed to explore diet-induced inflammation after consecutive wholegrain rye and refined wheat-based meals.

## Methods

### Study design, participants, and intervention diets

**FIGURE 1:**
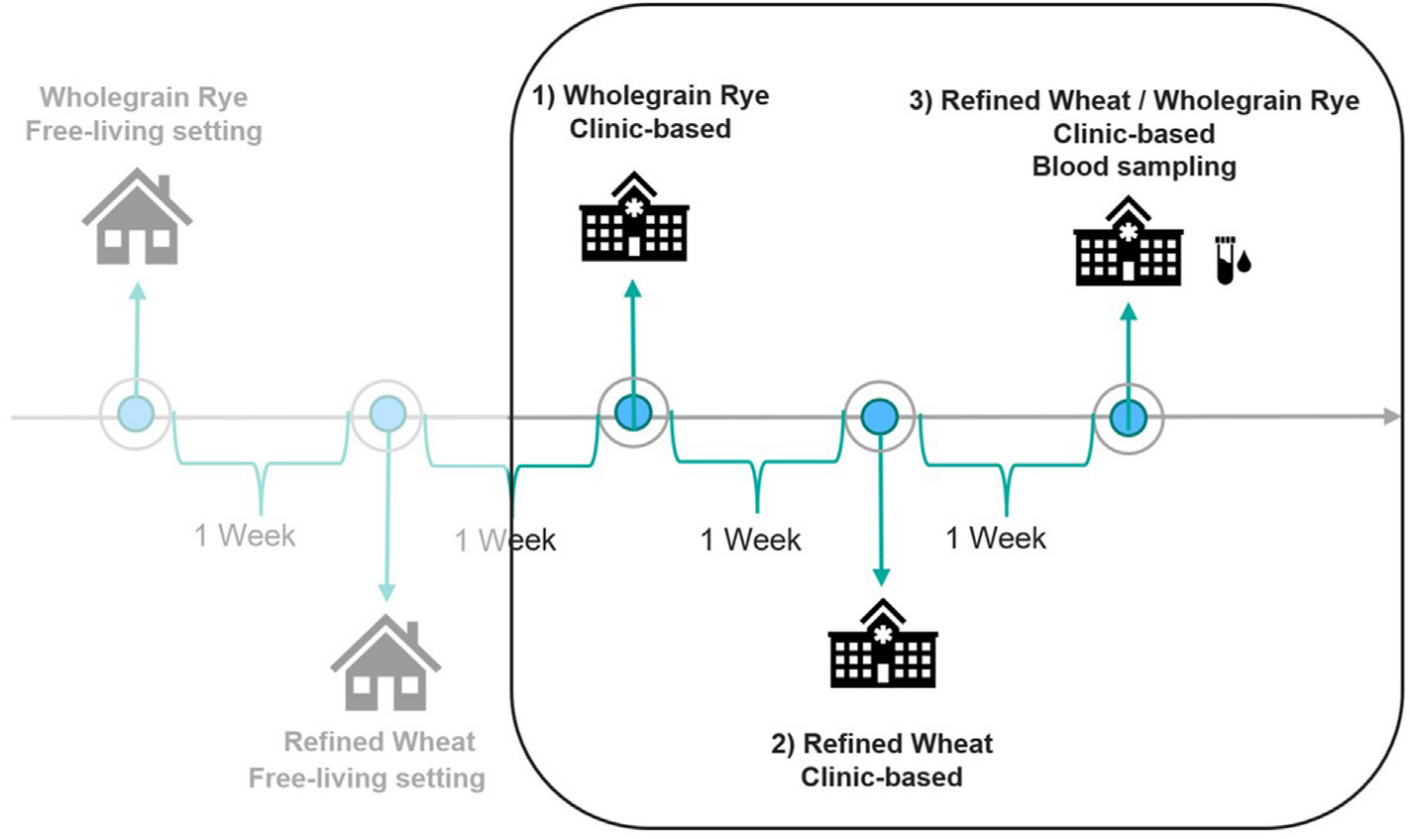
Overview of the study design and interventions reported in this paper. Intervention days 1–3 were all conducted at the research clinic. On intervention day 1, participants adhered to a wholegrain rye-based diet; on intervention day 2, participants adhered to a refined wheat-based diet; on intervention day 3, participants were randomly assigned to either a rye or wheat-based diet (50:50), with venous blood samples drawn throughout the intervention day. Interventions were conducted in random order.

A randomized crossover trial was conducted between August and November 2021 in Gothenburg, Sweden, with the main objective to evaluate self-reported appetite in free-living compared with clinic-based settings and to assess appetite response after wholegrain rye and reﬁned wheat-based diets [9]. Study details have been outlined elsewhere [9]. Secondary objectives of the trial, including the impact of the intervention diets on appetite-regulating gut hormones and glycemic control, were examined in this study. Briefly, 29 participants were randomly assigned to the sequence of 5 intervention days, each separated by 1-wk washout. This paper presents data from 3 intervention days conducted in a controlled clinical setting (Figure 1), whereas data from the 2 remaining intervention days in a free-living setting are not considered in this paper and have been reported elsewhere [9]. The sequence was generated using a Latin square design (“blockrand” package in R), kept in a closed, numbered envelope, and allocation was concealed at the time of participant enrollment. Randomization of intervention sequence was performed by a researcher not involved in the conduct of the study. Men and women aged 30–70 y with a BMI of 27–35 kg/m^2^ with blood pressure ≤160/105 mm Hg, fasting serum triglycerides ≤2.6 mmol/L, serum thyroid-stimulating hormone ≤3.7 mIE/L, hemoglobin ≥117g/L for women and ≥134 g/L for men, and plasma low-density lipoprotein ≤5.3 mmol/L were eligible for participation. Exclusion criteria included smoking or use of nicotine products, chronic gastrointestinal conditions, thyroid disorder, type 1 diabetes, medication for type 2 diabetes, or medication for weight management. For a full list of exclusion criteria, see Supplemental Text 1. Intervention days started at 08:00 and ended at 21:00. Participants followed hypocaloric meal plans providing 1300–2300 kcal/d, calculated based on their estimated energy requirements with a 500 kcal deficit. The energy requirements were determined using equations developed by Henry [20], assuming a physical activity level of 1.4. The full-day meal plan included a breakfast 08:00 (0 min) consisting of cereal puffs with milk, a lunch 12:00 (240 min) with tomato soup, crisp bread and cheese/jam, an afternoon snack 15:00 (420 min) consisting of crisp bread with cheese/jam and for dinner a goulash soup with soft bread and jam/cheese 19:00 (660 min). Participants consumed a fixed amount of wholegrain rye cereals during the first intervention day and corresponding refined wheat cereal products during the second intervention day. On the third intervention day, participants were randomly assigned (50:50) to either rye or wheat-based diets (Table 1). The rye and wheat cereals provided approximately one-third of the total energy and other foods were adjusted to meet the individual meal plan energy levels and contribute to a complete diet. The nutritional comparison of rye- and wheat-based intervention diets is presented in Table 1 and detailed nutritional data for the cereal products in Supplemental Table 1.

**TABLE 1:** Nutritional composition of rye- and wheat-based intervention diets

	Energy (kcal)	CHO^1^ (g)	Protein (g)	Fat (g)	Dietary fiber (g)^2^
(A) Wholegrain rye-based diet					
Whole day (0–780 min)	1697	210	66	56	32
Breakfast (0–240 min)	372	57	18	6	10
Lunch (240–420 min)	557	58	18	25	7
Snack (420–660 min)	184	22	7	5	2
Dinner (660–780 min)	584	74	24	21	12
(B) Refined wheat-based diet					
Whole day (0–780 min)	1708	219	72	58	9
Breakfast (0–240 min)	373	62	20	6	3
Lunch (240–420 min)	575	60	19	27	2
Snack (420–660 min)	190	22	7	5	1
Dinner (660–780 min)	571	76	26	20	3

Nutritional composition for intervention diets. Energy and macronutrient values are average values calculated from meal plans of all randomly assigned participants.

1 Available carbohydrates (CHO).

2 Dietary fiber is contributing with 2.0 kcal/g as described by FAO/WHO Expert Consultation on Carbohydrates in Human Nutrition 1997.

Throughout intervention days, participants answered questions about their perceived appetite (fullness, hunger and desire to eat) every 30 min from 08:00 to 12:00 and every 60 min from 13:00 to 21:00; see Supplemental Figure 1. Interstitial blood glucose was measured during all 3 intervention days through continuous glucose monitoring (CGM), whereas physical activity was monitored using pedometers throughout interventions. All 3 intervention days were conducted at the research clinic, where all meals were provided, and venous blood samples were drawn throughout the third intervention day (Supplemental Table 2). This study was prospectively registered at clinicaltrials.gov (NCT05004584) and approved by the Swedish Ethical Review Authority on May 11, 2021 (dnr 2021-02489). The study is reported in accordance with CONSORT [21].

### Continuous glucose monitors and physical activity

Interstitial blood glucose was measured during intervention days 1–3 through CGM (Abbott Freestyle Libre Pro IQ; Abbott Laboratories). The sensor was applied at the back of the upper part of the nondominant arm at the clinic and removed after completed interventions at the clinic by study personnel. Participants were blinded to glucose data. Physical activity was monitored throughout intervention days using pedometer (Yamax Digiwalker SW-700/701; Yamasa Tokei Keiki Co Ltd). Participants were instructed to attach the pedometer to the waistband at the hip in the morning 08:00 and the step count was registered at the end of each intervention day, at 21:00. Participants were instructed not to look at their step count during intervention days, to avoid behavioral changes.

### Blood sampling

On the third intervention day, half of the participants consumed wheat cereals, whereas the other half consumed rye cereals according to the diets at the clinic, and venous blood samples were drawn into 6 mL k_2_-EDTA and a 4 mL sodium heparin tubes by an experienced research nurse. Blood samples were collected 10 min before and 15, 35, 65, and 95 min after all meals, and additional sampling at 125, 155, 185, and 230 min depending on the proximity to the next meal. In total, 27 blood samples were drawn throughout the intervention day (Supplemental Table 2). An inhibitor cocktail was added to k_2_-EDTA tubes to inhibit protease degradation of peptide hormones. The inhibitor cocktail was prepared daily by dissolving 1 SIGMAFAST Protease Inhibitor tablet (catalog no. S8820; Sigma-Aldrich Co) in 2.2 mL deionized water containing 5.5 μL 10 mM-dipeptidyl peptidase-IV inhibitor KR-62436 (catalog no. K4264; Sigma-Aldrich) in dimethyl sulfoxide (catalog no. S8820; Sigma-Aldrich). The k_2_-EDTA and heparin tubes were kept on ice before and after sampling, and centrifuged at 4°C at 2500 relative centrifugal force (RCF) for 10 min. Plasma was transferred to cryotubes and stored at –20°C for a maximum of 7 d before being transferred to –80°C for long-term storage. Buffy coat and erythrocyte samples were extracted from a fasted k_2_-EDTA sample (time 0) and stored in –20°C for ≤7 d, before being transferred to –80°C for long-term storage.

### Blood analyses

Acyl-ghrelin (hereafter ghrelin), along with incretins GIP and active GLP-1, as well as total PYY was assayed by multiplex ELISA using electrochemiluminescence detection. Samples were thawed and vortexed, then analyzed in duplicate on 96-well multispot plates [Meso Scale Diagnostics (MSD)] with specific capture antibodies. Quantification of the immunoassay was conducted using the MSD imager (QuickPlex SQ120). Intra-assay variability was obtained through a quality control plasma sample on all plates and interassay variability was calculated from duplicate samples as percent coefficient of variation (CV%). Intra/ and interassay CV% were ghrelin 7.7/13.1; GIP 5.2/12.8; active GLP-1 10.8/26.6; and PYY 13.1/13.7. Concentrations were calculated from MSD software algorithm based on the luminescence signal, and standard curves were calculated separately for each plate/assay. Concentrations were calculated for 27 timepoints (whole-day response) and analyzed for postprandial periods after breakfast, lunch, snack, and dinner. Insulin and CRP were analyzed using accredited methods at the Clinical Chemistry Laboratory, Sahlgrenska University Hospital, Gothenburg. Plasma samples were analyzed for insulin by Abbott Laboratories ALINITY ci through immunoassay utilizing chemiluminescent detection (at 340 nm). CRP concentrations were obtained through turbidimetric immunoassay (at 572 nm), with Abbott Laboratories Alinity C clinical chemistry. Concentrations for insulin and CRP were calculated for 27 timepoints (whole-day response) and analyzed for postprandial periods. The interassay CV for CRP and insulin were 7% and 10%, respectively.

Inflammatory biomarkers GlycA, GlycB, and SPC were analyzed with proton NMR at Swedish Nuclear Magnetic Resonance Centre, Gothenburg. NMR data were acquired at 310K on a Bruker 600 MHz Avance III HD spectrometer equipped with a cooled SampleJet sample changer. Analysis algorithms (Bruker BioSpin) delivered lipoprotein profile and metabolite data and GlycA, GlycB, and SPC composite inflammation markers were extracted with an in-house MatLab script (available upon request) for 8 timepoints (whole-day response).

### Primary outcome measures

The primary outcome assessed to evaluate gut hormone responses was total area under the curve (tAUC), which was calculated, using approximate integrals according to the trapezoidal rule [22]. Postprandial glucose control was primarily evaluated through incremental area under the curve (iAUC) from CGM data using the trapezoidal rule. Additionally, mean concentrations of gut hormones and glucose were considered.

### Statistical analysis

The number of participants in the study was determined based on the primary aim of the trial, to detect 10% within-group differences in subjective appetite ratings; details have been previously published [9]. Complete case analysis was employed for all outcome measures, defining complete cases as participants who completed both rye- and wheat-based interventions at the research clinic for CGM-derived measures, and either a rye or wheat-based intervention with venous blood sampling for hormone and inflammation data. Measures of glycemic variability: SD, CV, mean amplitude of glycemic excursion (MAGE), and Peak Glucose Concentration (Cmax) were calculated from CGM. Glucose rise_0–2 h_ was defined as the maximum level above the baseline within the 2-h period, as a percentage of the average baseline level. Midpoint carry-forward imputation was utilized for hormone data, and in instances where the last observation was missing, the last observation carried-forward method was applied. Nonparametric missing value imputation using random forest (missForest, 1.4) was applied for CGM data before trapezoids, and iAUC was calculated.

We evaluated the effect of dietary interventions on whole-day glycemia and postprandial glycemic response: breakfast (0–240 min), lunch (240–420 min), snack (420–660 min), and dinner (660–780 min) in 2 separate mixed effects models for repeated measures accounting for intervention order. In model 1, glucose iAUC was evaluated as response variable and age, blood sampling, and step count were added as covariates. Participant was included as a random-effects variable and the interaction terms diet × intervention order and diet × step count were evaluated in separate models and nonsignificant interactions excluded from the final model. Model 1 was also used to analyze SD, MAGE, CV, and Cmax as response variables to assess glycemic variability. Model 2 was built to evaluate mean glucose concentration for all postprandial periods and included time as an additional covariate, after consideration of diet × time interactions.

A third mixed model was built to assess between-diet differences for all gut hormones and insulin in postprandial periods breakfast–dinner and whole-day contrast between rye- and wheat-based diets. Model 3 included covariates; age, step count, time, and participant were fitted as random-effects variables. Inflammatory biomarkers GlycA, GlycB, SPC, and CRP were analyzed in a modified model 3, including baseline concentration as covariate for the corresponding inflammatory response variable. Interaction terms: diet × step count and diet × time were considered. We assessed insulin and gut hormone tAUC as response variables in model 4, an analysis of covariance (ANCOVA) model including age and baseline concentration as covariates. Interaction effects of step count and diet were evaluated. In a sensitivity analysis, hormone data were normalized to baseline participants' baseline HOMA-IR, and tAUC was calculated and fitted to model 4. Associations between postprandial glucose iAUC, glucose rise_0–2 h_, subjective appetite, insulin, and gut hormones were investigated by Spearman rank correlation analysis.

Normality of residuals in all statistical models was assessed using Q–Q plots, while homoscedasticity was evaluated both graphically by plotting model residuals and quantitatively using Bartlett’s test. Data presented are estimated marginal means with SE of the mean and considered statistically significant at *P* < 0.05. All probability (*P*) values reported underwent post-hoc Bonferroni correction to take multiple comparisons into account. All statistical analyses were performed in R version 4.1.3 [packages: lme4 (1.1–35.1), dplyr (1.1.4), tidyverse (2.0.0), emmeans (1.10.0), ggeffects (1.5.0), lmetest (09–40), missForest (1.4), caTools (1.18.2), pracma (2.4.4), corrplot (0.92)].

## Results

**FIGURE 2:**
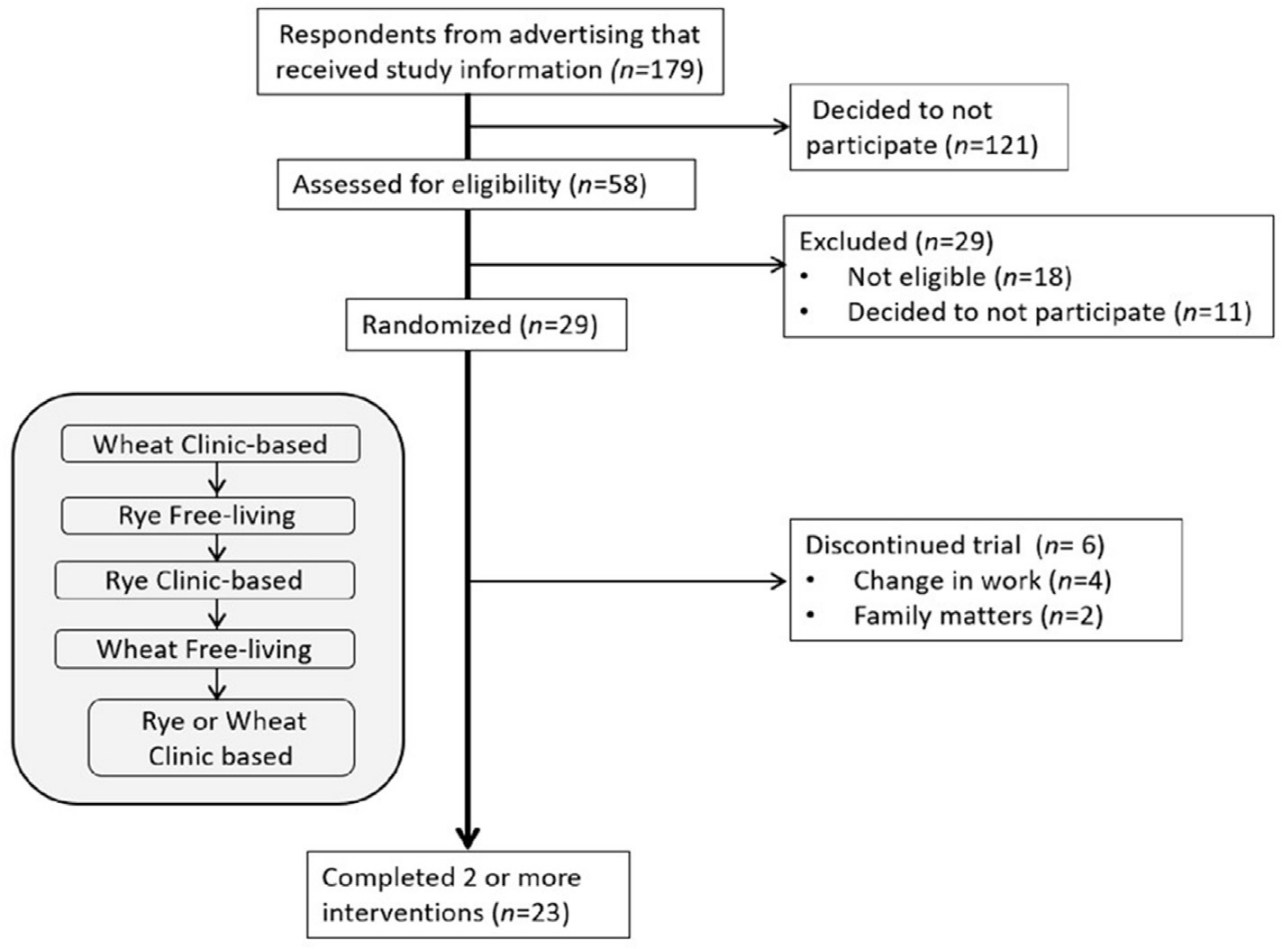
CONSORT flow diagram. Example of sequence order for interventions in figure.

In total, 58 individuals were screened for eligibility to participate in the study (Figure 2) and 29 participants, 56 ± 13 y, predominantly female (*n* = 16) were randomly assigned to intervention order (Table 2). No adverse events were reported. Twenty-one study participants completed interventions 1 and 2 at the research clinic (Table 2) and 21 participants completed the third clinic-based intervention with continuous blood sampling, 9 of which were randomly assigned to rye and 12 to the wheat-based diet (Table 3). Because of substantial deviations from the study protocol, 1 participant assigned to the wheat-based diet was excluded from analysis for not consuming the intervention foods.

**TABLE 2:** Baseline characteristics of participants (total and completers)

	Total (*n* = 29)	Completers (intervention 1 and 2) (*n* = 21)	Completers (intervention 3) (*n* = 20)
Female (%)	72	76	73
Age	55.2 ± 12.8	56.0 ± 12.9	56.5 ± 12.6
Weight	86.4 ± 12.8	87.7 ± 13.7	85.7 ± 9.5
BMI	29.7 ± 2.3	30.0 ± 2.4	30.1 ± 1.9
Systolic BP	130.6 ± 12.9	130.9 ± 12.6	131.5 ± 12.6

Abbreviation: BP, blood pressure.

Data are presented as means ± SD.

**TABLE 3:** Baseline characteristics of participants that completed the third intervention

Completers: intervention 3		
	Rye (*n* = 9)	Wheat (*n* = 11)
Female (%)	67	73
Age	58.9 ± 10.5	56.5 ± 12.6
Weight	92.0 ± 18.0	85.7 ± 9.5
BMI	30 ± 2.9.0	30.1 ± 1.9
Systolic BP	132.8 ± 10.2	131.5 ± 12.6
HOMA-IR^1^	4.7 ± 6.3	1.9 ± 1.0

Abbreviation: BP, blood pressure.

Data are presented as means ± SD.

1 Outlier identified in the rye-group.

In the analysis of GLP-1 data, 5 participants were excluded where missing data exceeded 30%, and 1 participant with fasting GLP-1 levels 10 times higher than the median was identified as an outlier and subsequently excluded from analysis. Insufficient liver dipeptidyl peptidase-4 and subsequent disrupted incretin hormone breakdown were suggested as reason for elevated fasting GLP-1, as consulted with an endocrinology expert. Hence, 14 participants (5 rye and 9 wheat) with <3% missing data, before imputation were included in the final analysis of GLP-1. PYY data were excluded from analysis because of considerable background noise in the assay, with over a third of the observations falling below the limit of detection. For GIP and ghrelin, all participants were included in the analysis and missing data accounted for 2.4% and 4.4%, respectively, before imputations. Complete case analysis was performed for glucose data derived from CGM, resulting in 21 participants who completed both rye- and wheat-based dietary interventions in the research clinic being included in the analysis (Table 2). Missing CGM data accounted for 4% before imputation. No statistically significant interaction with intervention order and diet was found and interaction terms were removed in the final model. Inflammatory markers GlycA, GlycB, SPC, and CRP had missing data 0.5%–1.3% and were all analyzed without imputation.

### Overall, no differences in whole-day gut hormone concentrations between wholegrain rye and refined wheat diets

**FIGURE 3:**
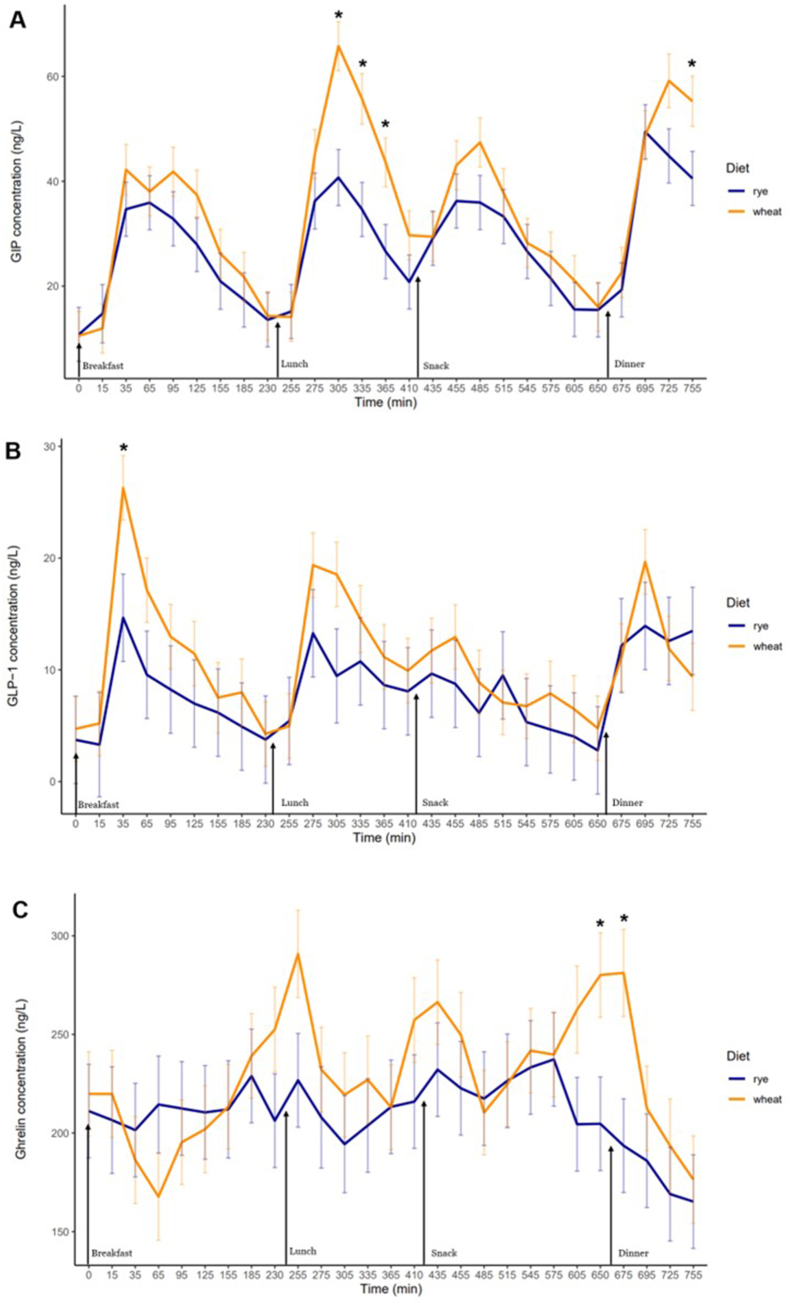
Gut hormone responses: GIP (A), GLP-1 (B) and ghrelin (C) measured as mean concentrations for rye- and wheat-based diets 0–755 min. Significant differences between diets at certain timepoints are indicated by **∗***P* < 0.05. Data are presented as estimated marginal means ± SEM, *n* = 20. GIP, glucose-dependent insulinotropic peptide; GLP-1, glucagon-like peptide-1.

No differences in GIP, ghrelin, or GLP-1 were found between the diets when measured throughout the whole intervention day as tAUC (Table 4). However, GIP tAUC after the rye-based lunch was 31% (*P* < 0.05) lower compared with the wheat-based lunch. Moreover, mean ghrelin concentrations were 29% (*P* < 0.05) lower after rye-based dinners compared with those based on wheat (Figure 3C), aligning with a 23% (*P* = 0.08) reduction in ghrelin tAUC. No differences between diets were observed in plasma insulin concentrations throughout the intervention day or after specific meals.

**TABLE 4:** Postprandial gut hormone response

Measure	Wholegrain rye	Refined wheat	*P* value difference between diets
Whole-day GLP-1	6146 ± 1726	8181 ± 1273	0.37
Breakfast GLP-1	1723 ± 597	2674 ± 440	0.24
Lunch GLP-1	1657 ± 438	2280 ± 323	0.29
Snack GLP-1	1553 ± 405	1937 ± 299	0.47
Dinner GLP-1	1213 ± 399	1290 ± 294	0.88
Whole-day GIP	20,915 ± 2698	26,205 ± 2438	0.17
Breakfast GIP	5633 ± 643	7251 ± 581	0.08
Lunch GIP	5168 ± 800	7497 ± 723	0.047**∗**
Snack GIP	6301 ± 909	7402 ± 822	0.38
Dinner GIP	3774 ± 579	4515 ± 523	0.36
Whole-day ghrelin	15,9514 ± 12,185	17,4014 ± 10,991	0.40
Breakfast ghrelin	49,884 ± 2816	47,342 ± 2540	0.52
Lunch ghrelin	38,189 ± 3902	42,586 ± 3520	0.42
Snack ghrelin	52,812 ± 4816	59,990 ± 4344	0.29
Dinner ghrelin	18,629 ± 2148	24,096 ± 1937	0.08

Postprandial gut hormone responses; glucose-dependent insulinotropic peptide (GIP), ghrelin and glucagon-like peptide-1 (GLP-1) measured as total AUC for rye- and wheat-based diets in postprandial periods: whole day 0–755 min, breakfast 0–230 min, lunch 240–410 min, snack 420–650, and dinner 660–755 min. Significant difference between diets in the same postprandial period is indicated by ∗*P* < 0.05. Data are presented as estimated marginal means ± SEM, *n* = 20.

Baseline fasting mean insulin was higher in the rye group and mean HOMA-IR was calculated to 4.7 in the rye group compared with 1.9 in the wheat group (Table 3). In a sensitivity analysis, gut hormone tAUC data were normalized to participants' baseline HOMA-IR and corresponding ANCOVA models were fitted. When taking differences in HOMA-IR into account, the results changed considerably for GLP-1, indicating a 61% (*P* = 0.015) lower whole-day GLP-1 response to the rye-based diet compared with the wheat-based diet, as measured by tAUC. A similar difference between diets was observed for breakfast, lunch, and snack postprandial periods (Supplemental Table 3). GIP whole-day tAUC was also 40% (*P* = 0.03) lower after the rye-based compared with wheat-based diet, with similar reductions observed during the lunch and snack postprandial periods. Ghrelin tAUC was 33% (*P* = 0.04) lower after the rye-based dinner but no difference measured over the whole day.

### Wholegrain rye compared with refined wheat foods improved measures of glycemia

**FIGURE 4:**
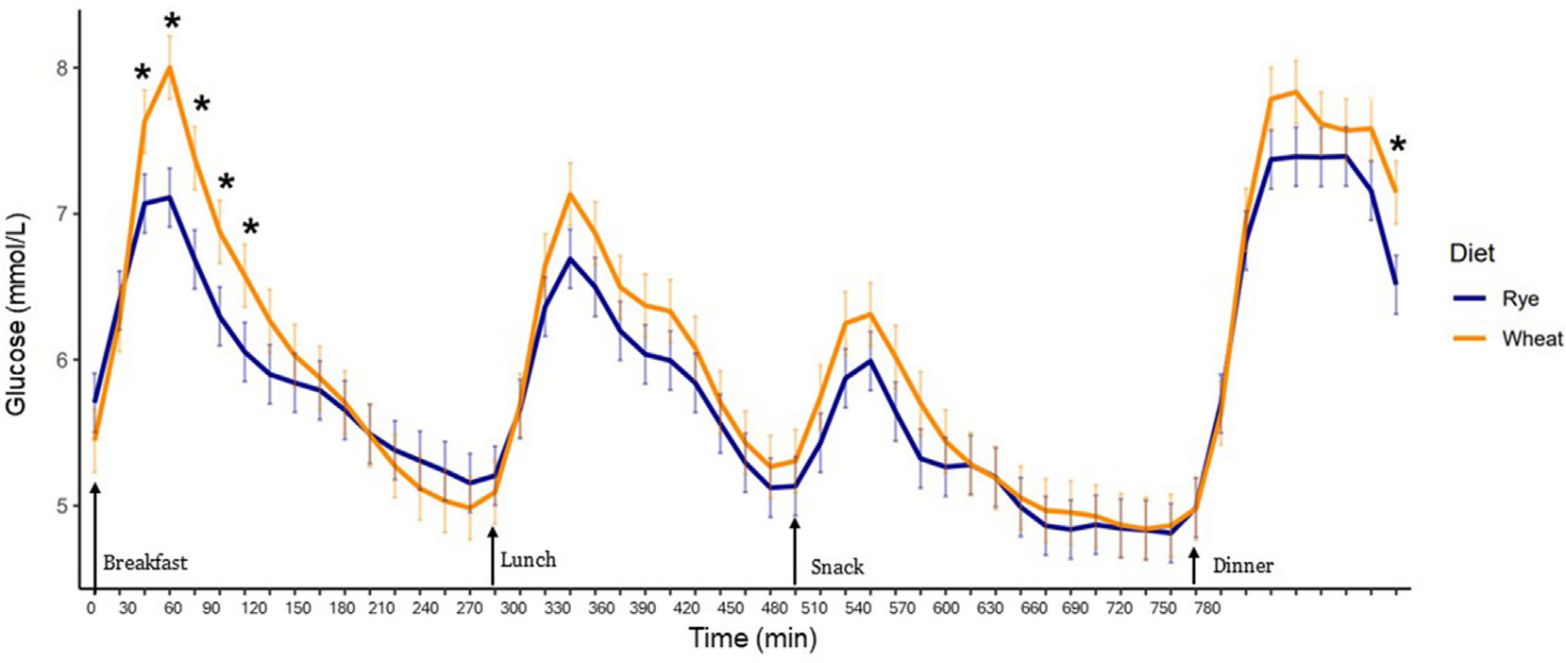
Glucose concentrations over the whole day 0–780 min after rye- and wheat-based diets. Significant difference between diets at certain timepoints are indicated by ∗*P* < 0.05. Data are presented as estimated marginal means ± SEM, *n* = 21.

The wholegrain rye-based diet resulted in 30% (*P* < 0.001) lower whole-day glucose iAUC as compared with the wheat-based diet (Table 5). The reduction in glycemic response to the rye-based diet was most pronounced in the morning (Figure 4) and early afternoon with 28% (*P* < 0.001) lower glucose iAUC after the breakfast and 26% (*P* = 0.02) after the lunch (Table 5). Day-long glycemic variability was also lower for rye compared with wheat-based diets when measured as SD [–0.13 mmol/L (95% confidence interval (CI): –0.7, –0.19)] and Cmax [–0.75 mmol/L (95% CI: –0.57, –0.93)] (Table 6). No difference between diets was observed for day-long CV and MAGE. After breakfast, a reduction in Cmax was observed [–0.58 mmol/L (95% CI: –0.35, –0.81)], and a similar decrease was observed after the snack [–0.46 mmol/L (95% CI: –0.33, –0.59)]. However, no significant difference was detected between diets after lunch and dinner.

**TABLE 5:** Postprandial glucose responses—iAUC calculated from CGM

Postprandial period	Rye	Wheat	*P* value difference between diets
Whole day	460 ± 58.4	657 ± 62.8	0.0001**∗∗**
Breakfast	143 ± 21.7	198 ± 22.5	<0.0001**∗∗**
Lunch	147 ± 22.3	198 ± 25.0	0.019**∗**
Snack	73.9 ± 13.4	74.5 ± 16.1	0.97
Dinner	245 ± 22.1	285 ± 25.2	0.084

Abbreviations: CGM, continuous glucose monitoring; iAUC, incremental AUC.

Postprandial glucose responses measured as iAUC. Significant differences between diets in the same postprandial period are indicated by ∗*P* < 0.05 and ∗∗*P* < 0.005. Data are presented as estimated marginal means ± SEM, *n* = 21.

**TABLE 6:** Measures of glycemic variability calculated from CGM

Measures of glycemic variability		Rye	Wheat	*P* value difference between diets
Whole-day	CV	18.5 ± 1.24	20.0 ± 1.34	0.13
Whole-day	SD	1.08 ± 0.09	1.21 ± 0.09	0.04**∗**
Whole-day	MAGE	0.33 ± 0.03	0.38 ± 0.03	0.06
Whole-day	Cmax	8.61 ± 0.22	9.37 ± 0.24	0.0004**∗∗**
Breakfast	Cmax	7.82 ± 0.33	8.4 ± 0.35	0.019**∗**
Lunch	Cmax	7.44 ± 0.22	7.84 ± 0.25	0.11
Snack	Cmax	6.43 ± 0.16	6.89 ± 0.18	0.002**∗∗**
Dinner	Cmax	7.63 ± 0.3	7.96 ± 0.33	0.13

Abbreviation: CGM, continuous glucose monitoring.

Measures of glycemic variability; coefficient of variation (CV), maximum concentration (Cmax), SD, and mean amplitude of glycemic excursion (MAGE) for rye- and wheat-based diets. Significant difference between diets in the same postprandial period is indicated by ∗*P* < 0.05 and ∗∗*P* < 0.005. Data are presented as estimated marginal means ± SEM, *n* = 21.

### Postprandial glucose and insulin correlated with hunger and satiety

We found that postprandial glucose iAUC_0-4h_ was correlated with fullness_0–4h_ after breakfast (*r* = 0.44, *P* = 0.002). Additionally, inverse correlations were found for hunger_0–4h_ (*r* = –0.40, *P* < 0.004) and desire to eat_0–4h_ (*r* = –0.38, *P* < 0.006) measured as tAUC (Table 7). Postprandial glucose rise_0–2h_ was correlated with fullness_0–4h_ (*r* = 0.55, *P* = 0.008) and inversely correlated with hunger_0–4h_ (*r* = –0.57, *P* = 0.006) and desire to eat_0–4h_ (*r* = –0.63, *P* = 0.002) after the wheat-based breakfast (Supplemental Figure 2B). In addition, for the wheat-based breakfast, insulin tAUC_0–4h_ was correlated with fullness_0–4h_ (*r* = 0.75, *P* = 0.03) and inversely correlated with hunger_0–4h_ (*r* = –0.8, *P* = 0.01) and desire to eat_0–4h_ (*r* = –0.77, *P* = 0.02) (Table 7). Irrespective of diet, insulin tAUC_0–3h_ after lunch, was correlated with fullness_0–3h_ and inversely correlated with hunger_0–3h_ and desire to eat_0–3h_ (Table 7). However, significant associations with GIP were observed only in response to wheat-based meals. GIP_0–4h_ after the wheat-based breakfast showed an inverse correlation with hunger_0–4h_ (*r* = –0.83, *P* = 0.02), and GIP_0–3h_ after the wheat-based lunch with desire to eat_0–3h_ (*r* = –0.77, *P* = 0.02) (Supplemental Figure 2B). Interestingly, GLP-1_0–4h_ after the wheat-based snack meal was inversely correlated (*r* = –0.86, *P* = 0.02) with glucose iAUC_0–4h_ (Supplemental Figure 2B). No associations were observed for ghrelin and GLP-1 and subjective appetite measures.

**TABLE 7:** Postprandial insulin and glucose and associations with subjective appetite measures for rye- and wheat-based meals

	Hunger tAUC	Desire to eat tAUC	Fullness tAUC
Breakfast 0–4 h			
Glucose rise 0–2 h	*r* = –0.46, *P* < 0.001**∗∗**	*r* = –0.46, *P* < 0.001**∗∗**	*r* = 0.45, *P* = 0.001**∗∗**
Glucose iAUC 0–4 h	*r* = –0.40, *P* < 0.004**∗∗**	*r* = –0.38, *P* = 0.006**∗**	*r* = 0.44, *P* < 0.002**∗∗**
Insulin tAUC 0–4 h	*r* = –0.51, *P* = 0.01**∗**	*r* = –0.52, *P* = 0.028**∗**	*r* = 0.59, *P* = 0.03**∗**
Insulin rye^1^	*r* = –0.50, *P* = 0.18	*r* = –0.55, *P* = 0.13	*r* = –0.55, *P* = 0.13
Insulin wheat^1^	*r* = –0.8, *P* = 0.01**∗**	*r* = –0.77, *P* = 0.02**∗**	*r* = 0.75, *P* = 0.03**∗**
Lunch 0–3 h			
Glucose rise 0–2 h	*r* = –0.15, *P* = 0.34	*r* = –0.06, *P* = 0.7	*r* = 0.12, *P* = 0.45
Glucose iAUC 0–3 h	*r* = –0.19, *P* = 0.21	*r* = 0.12, *P* = 0.45	*r* = 0.22, *P* = 0.15
Insulin tAUC 0–3 h	*r* = –0.58, *P* = 0.02**∗**	*r* = –0.52, *P* = 0.03**∗**	*r* = 0.75, *P* < 0.001**∗∗**
Snack 0–4 h			
Glucose rise 0–2 h	*r* = –0.16, *P* = 0.28	*r* = –0.13, *P* = 0.36	*r* = 0.15, *P* = 0.31
Glucose iAUC 0–4 h	*r* = –0.22, *P* = 0.12	*r* = –0.24, *P* = 0.09	*r* = 0.2, *P* = 0.16
Insulin tAUC 0–4 h	*r* = –0.29, *P* = 0.23	*r* = –0.30, *P* = 0.22	*r* = 0.43, *P* = 0.068
Dinner 0–2 h			
Glucose rise 0–2 h	*r* = 0.07, *P* = 0.66	*r* = 0.02, *P* = 0.92	*r* = –0.02, *P* = 0.92
Glucose iAUC 0–2 h	*r* = –0.19, *P* = 0.21	*r* = –0.32, *P* = 0.04**∗**	*r* = 0.19, *P* = 0.23
Insulin tAUC 0–2 h	*r* = –0.34, *P* = 0.18	*r* = –0.38, *P* = 0.14	*r* = 0.15, *P* = 0.56

Abbreviations: iAUC, incremental AUC; tAUC, total AUC.

Spearman rank correlation (*r*) between postprandial glucose rise, glucose iAUC, insulin tAUC and subjective appetite measures: fullness, desire to eat and hunger measured as tAUC, across diets.

1 Insulin tAUC_0–4 h_ after rye- and wheat-based breakfasts. Significant correlations are indicated by ∗*P* < 0.05 and ∗∗*P* < 0.005. *n* = 21.

### Consecutive wholegrain rye-based meals elevate GlycA levels without affecting CRP

We observed higher GlycA and SPC after the rye-based diet compared with the wheat-based diet measured as mean z-scores over the whole day (Table 8). Additionally, GlycB was trending in the same direction with higher z-scores 0.35 ± 0.17 (*P* = 0.054) after the rye-based diet. GlycA increased above fasting concentrations with a mean increase of 10.7% in the rye group and 6.2% in the wheat group (Supplemental Table 4). We observed no difference in CRP concentrations between diets.

**TABLE 8:** Continuously measured inflammatory markers in plasma

	Z-score difference between diets ± SE	*P* value difference between diets
GlycA	0.36 ± 0.13	*P* = 0.014**∗**
GlycB	0.35 ± 0.17	*P* = 0.054
SPC	0.27 ± 0.13	*P* = 0.044**∗**
CRP	0.024 ± 0.18	*P* = 0.89xx

Abbreviations: CRP, C-reactive protein; GlycA, glycoprotein N-acetylation A; GlycB, glycoprotein N-acetylation B; SPC, supramolecular phospholipid composite peak.

Z-score difference of diet-induced inflammation measured over the whole day 0–725 min. Significant difference between diets is indicated by ∗*P* < 0.05. *n* = 20.

## Discussion

In this study, we evaluated the metabolic effects of substituting refined wheat with wholegrain rye foods as part of a complex diet, examining the day-long postprandial responses of incretin hormones, ghrelin, glucose, and inflammatory markers among men and women with overweight and obesity.

Replacing refined wheat cereals with wholegrain rye cereals within a complex diet had no effect on GIP, GLP-1, and ghrelin concentrations throughout the whole day. The rye-based dinner reduced mean postprandial ghrelin concentrations compared with the wheat-based dinner. Additionally, the wholegrain rye-based diet reduced day-long glucose iAUC and improved measures of glycemic variability, indicating improved glycemic control. When adjusting for differences in baseline HOMA-IR in a sensitivity analysis, the wholegrain rye-based diet significantly reduced whole-day GLP-1 and GIP tAUC compared with the wheat-based diet. Interestingly, we observed increased GlycA and SPC after the rye-based diet, whereas CRP levels remained similar between diets.

Recently, we showed indications of reduced sensation of hunger and increased satiety during the afternoon and evening when free-living participants consumed consecutive meals based on wholegrain rye [9]. When we measured ghrelin concentrations as an objective marker of hunger in the same participants, levels were reduced after the rye-based dinner compared with the wheat-based dinner, in line with subjective reporting of reduced hunger and increased fullness [9]. Most studies comparing wholegrain rye with refined wheat have found increased postprandial self-reported satiety for wholegrain rye [[5], [6], [7],^10^]. Yet, few studies have investigated the effects on appetite-regulating hormones. In 2 acute meal studies by Rosén et al. [7,23], reduced postprandial ghrelin after intake of wholegrain rye compared with refined wheat products was found. In contrast, neither Hartvigsen et al. [5] nor Heinonen et al. [24] observed any difference in postprandial ghrelin response to wholegrain rye foods compared with refined wheat alternatives among participants with obesity. Hartvigsen et al. [5] also analyzed postprandial GLP-1 and showed significant reductions after wholegrain rye compared with semolina porridge. Juntunen et al. [11] demonstrated lower GLP-1 and GIP concentrations after wholegrain rye kernel bread compared with refined wheat bread, but no difference was measured as iAUC. Both studies employed a crossover design, focusing on the postprandial response to meals composed solely of rye products, unlike our study, which evaluates hormone response to diets containing approximately one-third of calories from rye intervention foods.

This study was initially designed to evaluate digital visual analogue scales in free-living compared with clinic-based settings and to assess subjective appetite response after wholegrain rye and reﬁned wheat-based diets and the assessment of gut hormone response in a parallel subset was a secondary objective. Participants who completed the third intervention with the rye-based diet demonstrated significantly higher baseline HOMA-IR compared with those after the wheat-based diet. This reflects progression toward insulin resistance and may influence glucose metabolism as well as incretin secretion. Additionally, interindividual variation in the hormone data proved to be higher than anticipated.

Hence, we opted to conduct a sensitivity analysis where incretin concentrations were normalized to the participants' baseline HOMA-IR. This analysis showed significantly lower whole-day GLP-1 and GIP response for the rye group compared with the wheat group. This trend was consistent across breakfast, lunch, and snack postprandial periods. These findings suggest a potential impact of wholegrain rye on incretin responses in individuals with overweight or obesity. However, the results from this sensitivity analysis should be interpreted with caution. Future studies investigating incretin and ghrelin response to complex meals and diets with wholegrain rye should consider a crossover design or stratified randomization reflecting biomarkers of insulin resistance.

In line with our study, systematic reviews and meta-analyses have shown improved postprandial glycemia with wholegrain cereals compared with refined alternatives [12,[25], [26], [27]]. However, few have investigated commonly consumed refined wheat cereals with wholegrain rye alternatives. Marventano et al. [25] showed reduced postprandial glucose iAUC for wholegrain rye foods compared with refined wheat; however, specifically, 3 studies by Rosén et al. [7,23,28] were driving the observed effect.

Recently, we conducted a narrative review [12], identifying the studies by Rosén et al. and additionally 2 studies showing reduced postprandial glucose AUC when comparing wholegrain rye with semolina porridge [5,10] and 1 study with soft pretzels as control [29]. Five studies investigated effects of replacing habitual cereals with wholegrain rye and found no difference in glycemic control [12]. Some studies reported decreased postprandial peak concentrations or sporadic observations after the consumption of rye-based foods [5,11,30,31], but most studies did not observe any effect of rye foods.

To our knowledge, this is the first study to show improved postprandial glycemia with consecutive meals with wholegrain rye compared with refined wheat cereals and not solely cereals but as part of a complex diet, constituting approximately one-third of the total caloric intake. Rosén et al. studied effects of specific wholegrain rye products on acute postprandial glycemia in young, healthy individuals, BMI <25 kg/m^2^, whereas Hartvigsen et al. examined glycemic response to rye porridge with added arabinoxylan and semolina porridge in adults with metabolic syndrome and mean BMI 31.3 kg/m^2^. Our findings align with those of Hartvigsen et al. for the breakfast postprandial period and they extend to improved day-long glycemia 0–780 min, under a mixed diet regime. Recently, microstructure examination of wholegrain rye products included in this study showed partially intact digesta particles after 120 min of digestion and less-degraded starch granules compared with refined wheat bread [32]. Additional in-vitro digestion analysis showed higher glucose and maltose release during digestion for wheat products. This may be attributed to increased viscosity of soluble arabinoxylans in rye, contributing to the improved glycemic profile we observed in our trial. The soluble arabinoxylans may also increase the viscosity of chyma, slowing gastric emptying and limiting glucose absorption rate in the small intestine.

In contrast to previous studies, we did not observe differences in plasma insulin between diets [12]. This may be due to previously mentioned differences in insulin between intervention groups at baseline. Interestingly, observed correlations between postprandial glucose rise_0–2h_, insulin and subjective appetite were limited to the wheat group. This trend extended to associations with GIP, demonstrating a positive correlation with fullness and an inverse correlation with hunger, among participants in the wheat group.

Wyatt et al. [33] recently showed that glucose dip_0–2h_ but not glucose rise_0–2h_ or glucose iAUC was correlated with self-reported hunger. In a meta-analysis, Flint et al. [34] suggest that postprandial insulin, but not glucose, is associated with increased satiety and decreased hunger in normal-weight individuals, as opposed to those who are overweight or obese. We report associations with insulin and self-reported appetite in line with Flint et al. [34] but we also observe similar associations with postprandial glucose in individuals with overweight and obesity. Furthermore, we observe that these associations are driven by wheat-based meals, where the postprandial response induces higher amplitude in insulin, glucose, and GIP concentrations compared with rye-based meals. Structural differences with slower degradation of starch from wholegrain rye cereals compared with refined wheat may influence the glucose absorption and observed differences in concentrations.

High intake of whole grains has been associated with lower CRP levels [35], although the results from intervention studies are inconclusive [13]. Recently, we showed reduced fasting CRP levels after a 12-wk dietary intervention rich in wholegrain rye compared with corresponding diet containing refined wheat cereals [36]. In this trial, we observed no differences in CRP concentrations. However, postprandial GlycA and SPC were elevated in the rye group. To date, studies connecting postprandial GlycA levels and traditional clinical markers are lacking. Wyatt et al. propose that postprandial GlycA may provide a more accurate reflection of an individual’s inflammatory status when compared with fasting GlycA and IL-6 levels [19]. We can speculate that the observed increase in GlycA is induced by rye foods, but the study was not primarily designed to evaluate GlycA, and results should be interpreted with caution. Further examination of inflammatory markers and oxidative stress in acute meals response is warranted in future studies.

Although this study comprised a carefully controlled feeding design, we focused on acute biomarkers and therefore cannot evaluate long-term health outcomes. In contrast to previous work, we assessed metabolic effects of successive meals where wholegrain rye foods contributed roughly one-third of the energy intake. This intervention mimics participants' habitual diet and may be sustainable over extended periods [36], and the observed metabolic effects could potentially contribute to long-term sustained health.

The study may have had limited power to detect effects of the wholegrain rye-based diet on incretin hormones, ghrelin, and inflammatory markers. Given the small sample size, these results should be interpreted with caution. Furthermore, the study was insufficiently powered to evaluate effects of the intervention diet in men and women separately, different levels of glycemic control, and progression toward insulin resistance. Hormonal fluctuations during the menstrual cycle may influence appetite control and eating behavior in menstruating women [37]. Information about menstruation and the use of hormonal contraceptives was not collected and controlled for, which may have inﬂuenced gut hormones, insulin, and glycemic response in female participants. With above-mentioned differences in metabolic response between individuals with normal weight and overweight/obesity, future studies should aim both BMI groups in the same study to enable stratified analyses of metabolic responses to wholegrain rye foods within a complex diet.

In conclusion, while our overall findings did not support the hypothesis that day-long levels of ghrelin and incretin hormones would decrease following consecutive wholegrain rye meals compared with refined wheat-based meals, we observed specific postprandial effects. The rye-based dinner reduced mean postprandial ghrelin concentrations, aligning with decreased hunger sensation after the same meal. Replacing refined wheat with wholegrain rye cereals within a complex diet reduced day-long glucose iAUC and measures of glycemic variability, indicating improved glycemic control in individuals with overweight and obesity. The rye-based diet induced higher levels of GlycA and SPC, which needs to be investigated further in acute meal response studies. Interestingly, postprandial insulin, GIP, and glucose rise_0–2 h_ were associated with sensations of satiety exclusively in the wheat-group, advocating for further studies investigating appetite regulation in individuals with overweight and obesity.

## Author contributions

The authors’ responsibilities were as follows – SÅ: conducted the study and collected the data; RL: was the guarantor of this work and, as such, had full access to all data in the study and took responsibility for the integrity of the data and the accuracy of the data analysis; SÅ, RL: conceived and designed the study, and critically revised the manuscript for intellectual content; DLW, SÅ: conducted immunoassays and analyzed gut hormones; SÅ: was responsible for data curation and statistical analysis; SÅ: drafted the manuscript; SÅ, RL, PMH, EN: revised and edited the manuscript; and all authors: critically revised, read, and approved the final manuscript.

## Data availability

Data described in the manuscript, code book, and analytic code will be made available upon request pending (e.g., application and approval, payment, other).

## Funding

RL received grants from government research council Formas under grant number 00542, 2014 and the APC was funded by Swedish Research Council under grant number 2017-05840. RL also received funding for this work from Lantmännen Research Foundation, Lantmännen Ek and Barilla who provided rye and wheat intervention products. RL is the initiator of the Nordic Rye Forum, which is a Nordic collaboration platform between academia, institutes, and enterprises with interest in rye and health. The Nordic Rye Forum obtains annual fees from industrial partners to fund the activities.

## Conflict of interest

RL is an Editor for The Journal of Nutrition and played no role in the journal's evaluation of the manuscript. None of the authors reported a conflict of interest related to the study.
