# Supplementary material for: Postprandial Effects of Four Test Meals Containing Wholegrain Rye or Refined Wheat Foods on Circulating Incretins, Ghrelin, Glucose, and Inflammatory Markers
Source: J Nutr. 2024 Nov 6;155(1):185–96. doi: 10.1016/j.tjnut.2024.10.046 (PMC11795698; doi:10.1016/j.tjnut.2024.10.046)
Supplement: Multimedia component 1 [file mmc1.docx]

Supplementary Material:

**Supplementary Text 1.** Exclusion criteria

- Blood donation or participation in a clinical study with blood sampling within 30 days prior to screening visit and throughout the study
- Using nicotine products on a daily basis (incl. chewing gum, patches, snus etc.)
- Using e-cigarettes (regardless of nicotine content)
- Following any weight reduction program or having followed one during the last 6 months prior to screening.
- Diastolic blood pressure ≥ 105 mm Hg at screening
- Systolic blood pressure ≥ 160 mm at screening
- History of stomach or gastrointestinal conditions (Inflammatory bowel disease, Crohn’s disease, malabsorption, colostomy, bowel resection, gastric bypass surgery etc.)
- More than 10 hours physical activity per week (e.g. sport, fitness or similar). Persons with high BMI due to high level of fitness (normal/low body fat in combination high muscle mass) will not be included in the study, as such person will not be classified as overweight despite high BMI. In case of doubt regarding the classification of a potential participant, the medical doctor will be consulted.
- History of heart failure or heart attack within 1 year prior to screening
- Having type-I diabetes
- Receiving pharmacological treatment for type-II diabetes (treatments based on life-style interventions are acceptable, as long as they are compatible with the study protocol)
- Previous gastrointestinal surgery, with the exception of minor surgeries such removal of appendix or gall bladder at least 6 months prior to screening.
- Thyroid disorder
- History of drug or alcohol abuse
- Stroke or transient ischemic attack (TIA) within 1 year prior to screening
- Consumption of drugs aimed at weight management or drugs affecting body weight to a degree that is considered unsuitable for study participation by responsible physician.
- Pregnant, lactation or planning a pregnancy within the timeframe of the study. Pregnancy must have ended at least 6 months prior to screening, and lactation must have ended at least 1 month prior to screening.
- Food allergies, intolerances or dietary restrictions (e.g. vegetarian) preventing consumption of any products included in the study
- Unable to sufficiently understand written and spoken Swedish to provide written consent and understand information and instructions from the study personal.
- Lack of suitability for participation in the study, for any reason, as judged by the medical doctor or PI.

**Supplementary Table 1.** Nutritional composition of rye and wheat intervention foods

| **(A)** | **Product weight (g)** | **Energy (kcal)** | **CHO (g)** | **Protein (g)** | **Fat (g)** | **Dietary fiber (g)^†^** | | |
| --- | --- | --- | --- | --- | --- | --- | --- | --- |
|  |  |  |  |  |  | *Total* | *Extr.* | *Unextr.* |
| Extruded rye puffs | 100.0 | 345.4 | 64.4 | 8.6 | 1.5 | 16.8 | 6.9 | 9.9 |
| Rye crispbread | 100.0 | 330.1 | 59.5 | 9.8 | 1.6 | 17.0 | 6.7 | 10.3 |
| Soft rye bread | 100.0 | 218.8 | 35.8 | 5.9 | 3.1 | 10.6 | 3.8 | 6.9 |
| Extruded wheat puffs | 100.0 | 369.2 | 73.0 | 12.4 | 1.3 | 5.4 | 2.5 | 2.9 |
| Wheat crispbread | 100.0 | 382.9 | 64.5 | 12.2 | 6.7 | 5.6 | 1.5 | 4.0 |
| Soft wheat bread | 100.0 | 266.9 | 48.8 | 9.9 | 2.4 | 3.53 | 1.03 | 2.5 |

| **(B)** | **Product weight (g)** | **Energy (kcal)** | **CHO (g)** | **Protein (g)** | **Fat (g)** | **Dietary fiber (g)^†^** | | |
| --- | --- | --- | --- | --- | --- | --- | --- | --- |
|  |  |  |  |  |  | *Total* | *Extr.* | *Unextr.* |
| Extruded rye puffs | 60 | 207.3 | 38.7 | 5.2 | 0.9 | 10.1 | 4.1 | 6.0 |
| Rye crispbread | 54 | 178.3 | 32.1 | 5.3 | 0.9 | 9.2 | 3.6 | 5.6 |
| Soft rye bread | 117 | 255.9 | 41.9 | 6.9 | 3.6 | 12.4 | 4.4 | 8.0 |
| Extruded wheat puffs | 60 | 207.4 | 43.8 | 7.4 | 0.8 | 3.3 | 1.5 | 1.7 |
| Wheat crispbread | 66 | 252.7 | 42.6 | 8.1 | 4.4 | 3.7 | 1.0 | 2.7 |
| Soft wheat bread | 72 | 192.2 | 35.1 | 7.1 | 1.7 | 2.5 | 0.7 | 1.8 |

(A) Nutritional composition per 100g of product, (B) nutritional composition for products throughout an intervention day.

† Dietary fibre is contributing with 2.0 kcal/g as described by FAO/WHO Expert Consultation on Carbohydrates in Human Nutrition 1997.

**Supplementary Figure 1.** Overview of intervention days with appetite questions and meal timings.


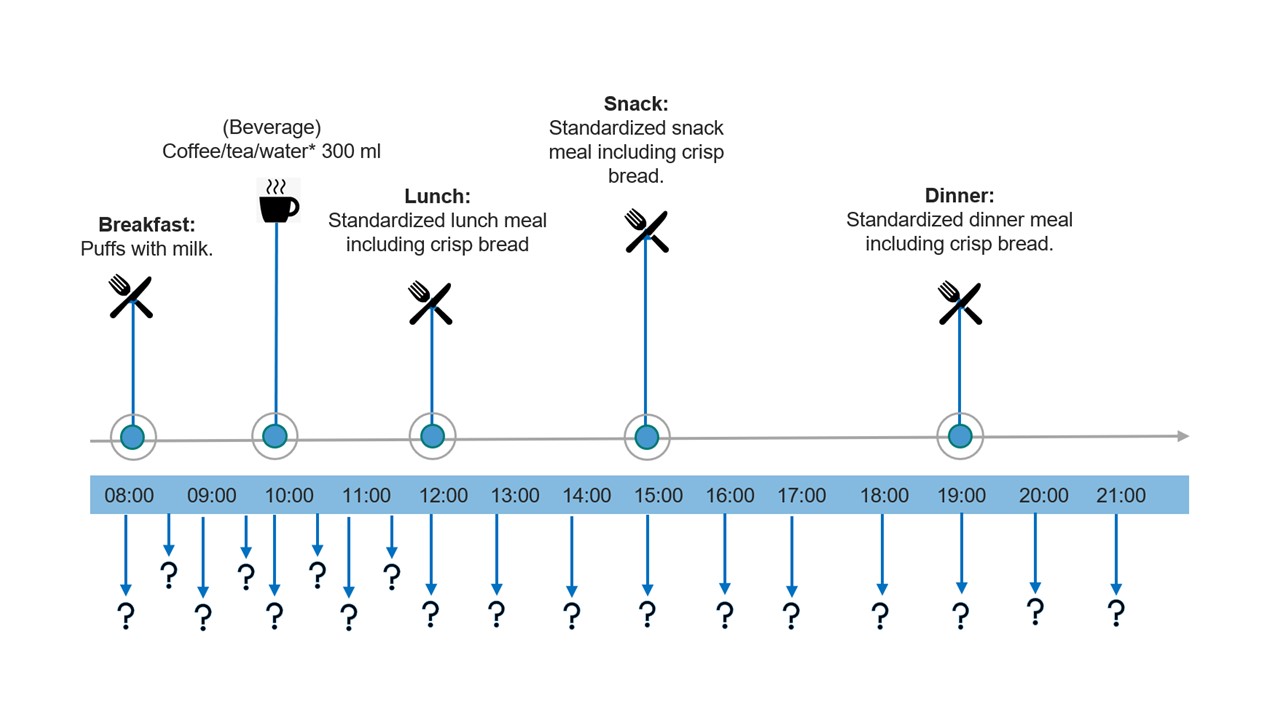


Question marks (?) indicate timings for VAS-questions; Fullness, Hunger and Desire to eat. *Study participants choose 300ml of coffee, tea or water and consume the same beverages for all intervention days. VAS: visual analogue scales.

**Supplementary Table 2.** Overview of blood sampling timepoints and meals

| **Time** | **Minutes** | **Meal** |
| --- | --- | --- |
| 07:50 | -10 |  |
| **08:00** | **0** | **Breakfast** |
| 08:15 | 15 |  |
| 08:35 | 35 |  |
| 09:05 | 65 |  |
| 09:35 | 95 |  |
| 10:05 | 125 |  |
| 10:35 | 155 |  |
| 11:05 | 185 |  |
| 11:50 | 230 |  |
| **12:00** | **240** | **Lunch** |
| 12:15 | 255 |  |
| 12:35 | 275 |  |
| 13:05 | 305 |  |
| 13:35 | 335 |  |
| 14:05 | 365 |  |
| 14:50 | 410 |  |
| **15:00** | **420** | **Snack** |
| 15:15 | 435 |  |
| 15:35 | 455 |  |
| 16:05 | 485 |  |
| 16:35 | 515 |  |
| 17:05 | 545 |  |
| 17:35 | 575 |  |
| 18:05 | 605 |  |
| 18:50 | 650 |  |
| **19:00** | **660** | **Dinner** |
| 19:15 | 675 |  |
| 19:35 | 695 |  |
| 20:05 | 725 |  |
| 20:35 | 755 |  |

All timepoints for venous blood sampling and meals throughout intervention day 3 with either rye or wheat-based diets.

**Supplementary Table 3.** Postprandial gut hormone response normalized to participants baseline HOMA-IR

| **Measure** | **Rye** | **Wheat** | **P-value difference between diets** | **Contrast** |
| --- | --- | --- | --- | --- |
| Whole-day GIP | 9138 ± 1944 | 15284 ± 1757 | 0.03* | -40% |
| Breakfast GIP | 2596 ± 601 | 4311 ± 543 | 0.05 | -40% |
| Lunch GIP | 2231 ± 513 | 4312 ± 463 | 0.01* | -48% |
| Snack GIP | 2651 ± 515 | 4255 ± 466 | 0.04* | -38% |
| Dinner GIP | 1640 ± 395 | 2638 ± 357 | 0.08 | -37% |
| Whole-day GLP-1 | 1732 ± 733 | 4449 ± 540 | 0.02* | -61% |
| Breakfast GLP-1 | 480 ± 288 | 1440 ± 212 | 0.03* | -67% |
| Lunch GLP-1 | 469 ± 210 | 1277 ± 155 | 0.01* | -63% |
| Snack GLP-1 | 439 ± 173 | 1048 ± 127 | 0.02* | -58% |
| Dinner GLP-1 | 343 ± 169 | 684 ± 125 | 0.14 | -50% |
| Whole-day Ghrelin | 76329 ± 13562 | 105936 ± 12233 | 0.13 | -28% |
| Breakfast Ghrelin | 24698 ± 3584 | 28573 ± 3233 | 0.44 | -14% |
| Lunch Ghrelin | 18512 ± 3856 | 26063 ± 3478 | 0.17 | -29% |
| Snack Ghrelin | 24611 ± 4622 | 36564 ± 4169 | 0.08 | -33% |
| Dinner Ghrelin | 8507 ± 1998 | 12735 ± 1802 | 0.04* | -33% |

Postprandial gut hormone responses; glucose-dependent insulinotropic peptide, (GIP), ghrelin and glucagon-like peptide-1 (GLP-1) measured as tAUC for rye- and wheat- based diets in postprandial periods: whole day 0–755 min, breakfast 0–230 min, lunch 240–410 min, snack 420–650, dinner 660–755 min. Significant difference between diets in the same postprandial period is indicated by *(p < 0.05). Data are presented as estimated marginal means ± SEM, n = 20.

**Supplementary Figure 2A**. Associations for rye-based meal responses: gut hormones, glucose and subjective appetite measures


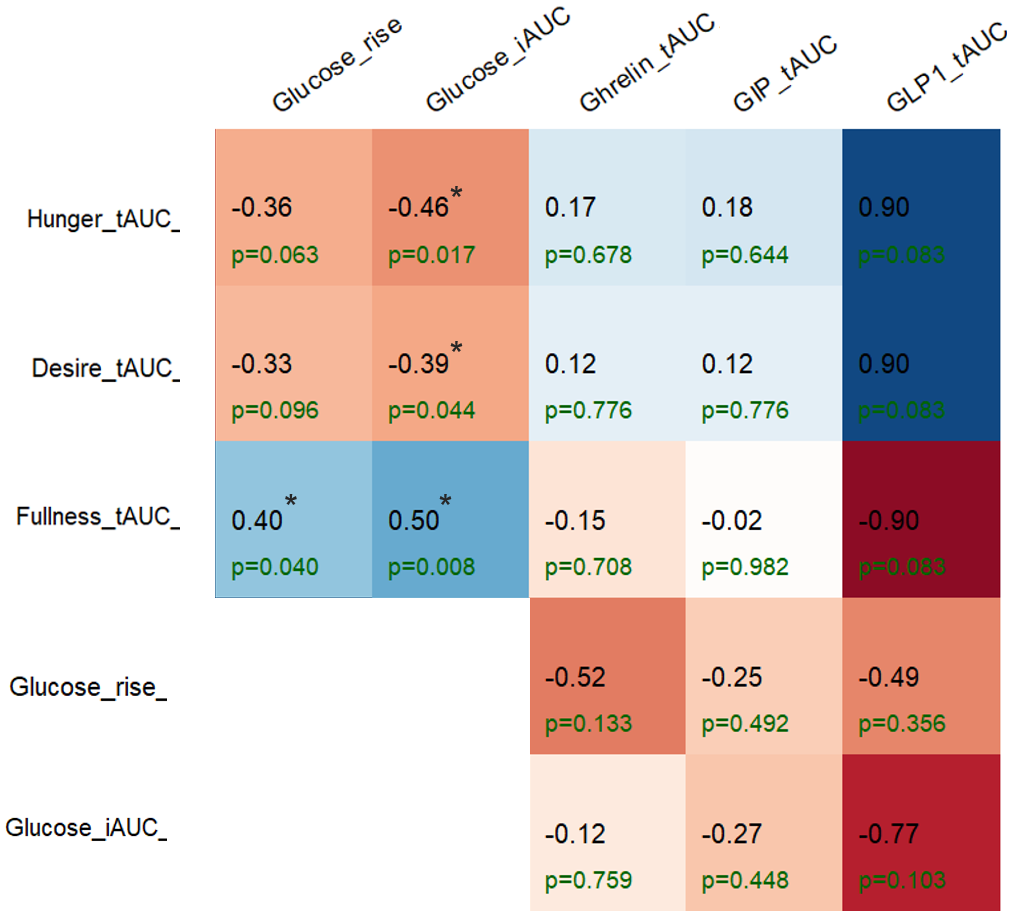

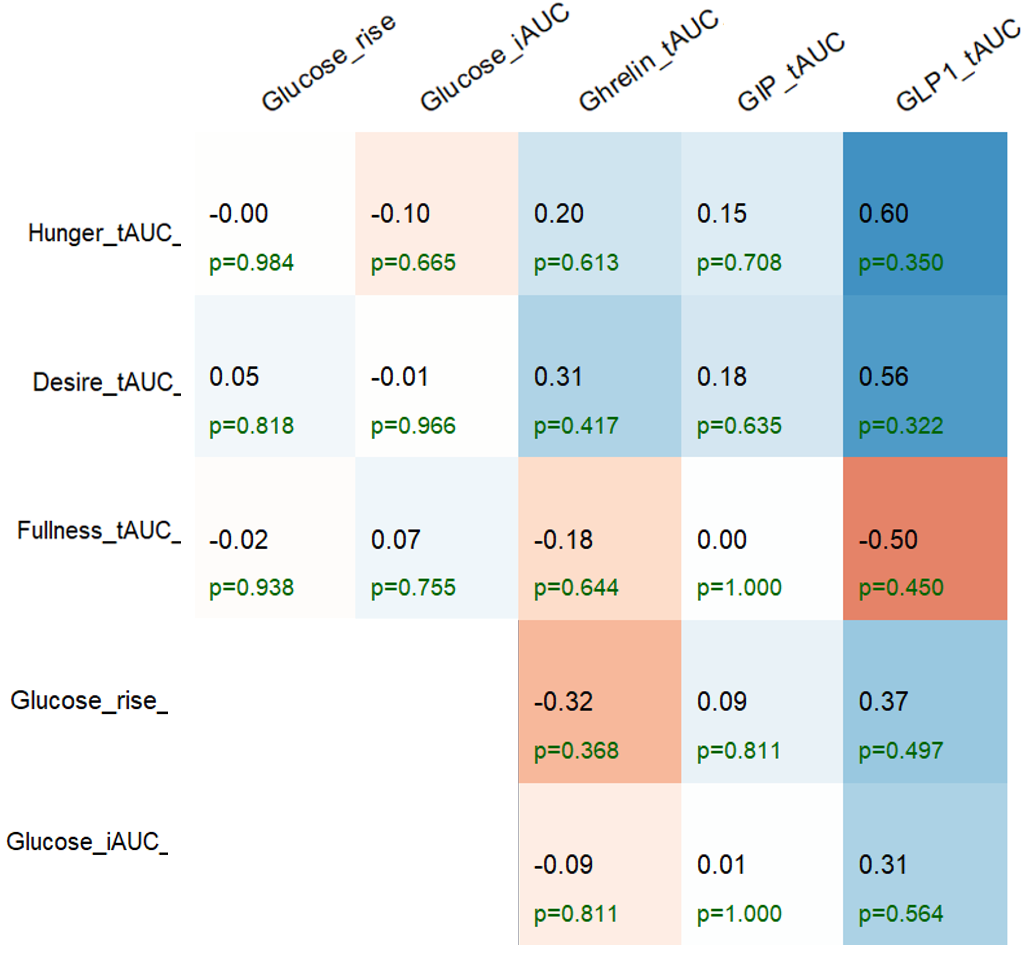


Breakfast 0 – 240min Lunch 240 – 420min


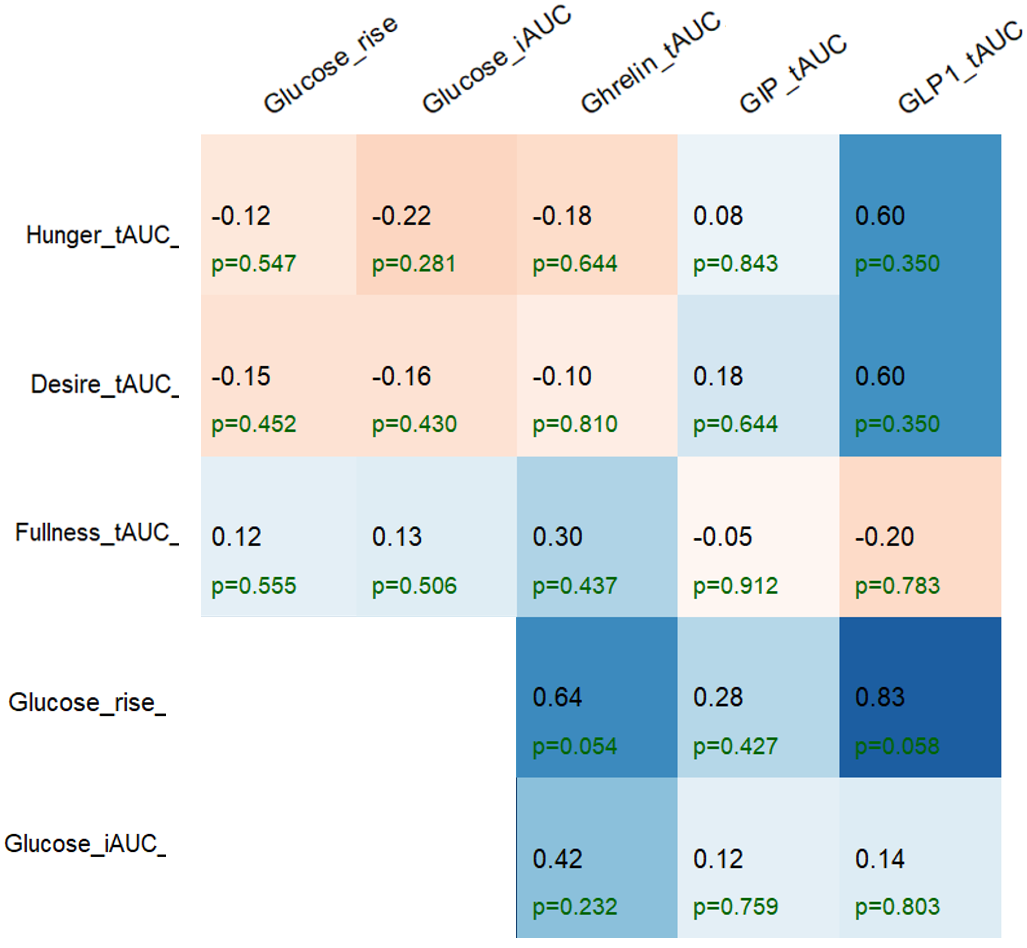

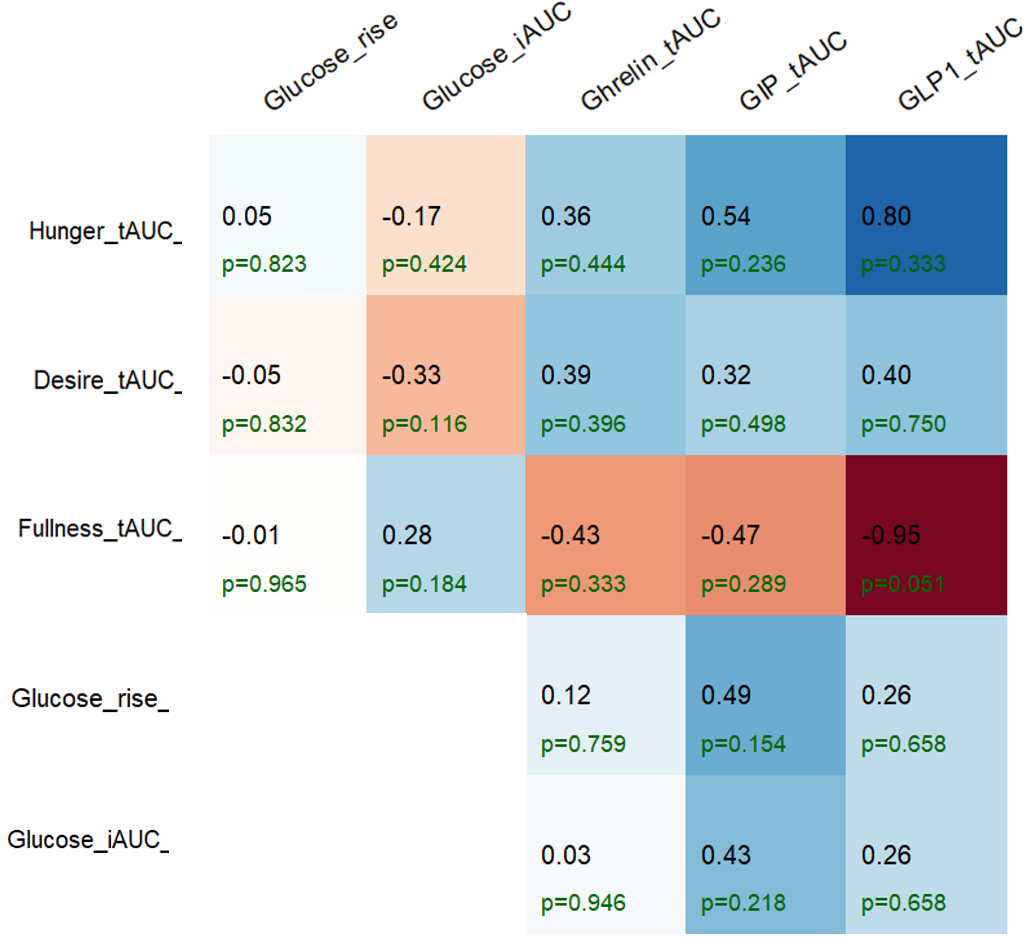


Snack 420 – 660min Dinner 660 – 780min

Spearman rank correlation (r) between postprandial glucose rise, glucose iAUC and hormones: Ghrelin, GIP and GLP-1 measured as tAUC, and subjective appetite measures: fullness, desire to eat and hunger measured as tAUC. Significant correlations are indicated by (p < 0.05) *, *n = 21*.

**Supplementary Figure 2B**. Associations for wheat-based meal responses: gut hormones, glucose and subjective appetite measures


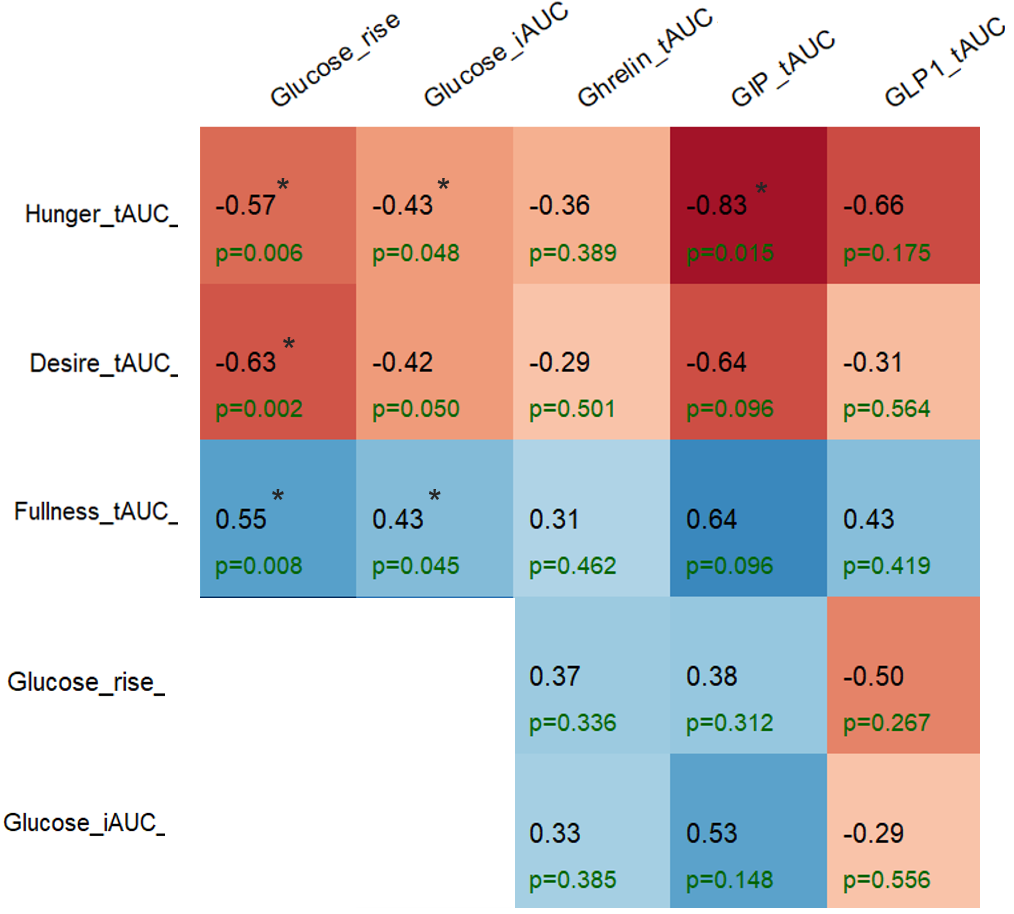

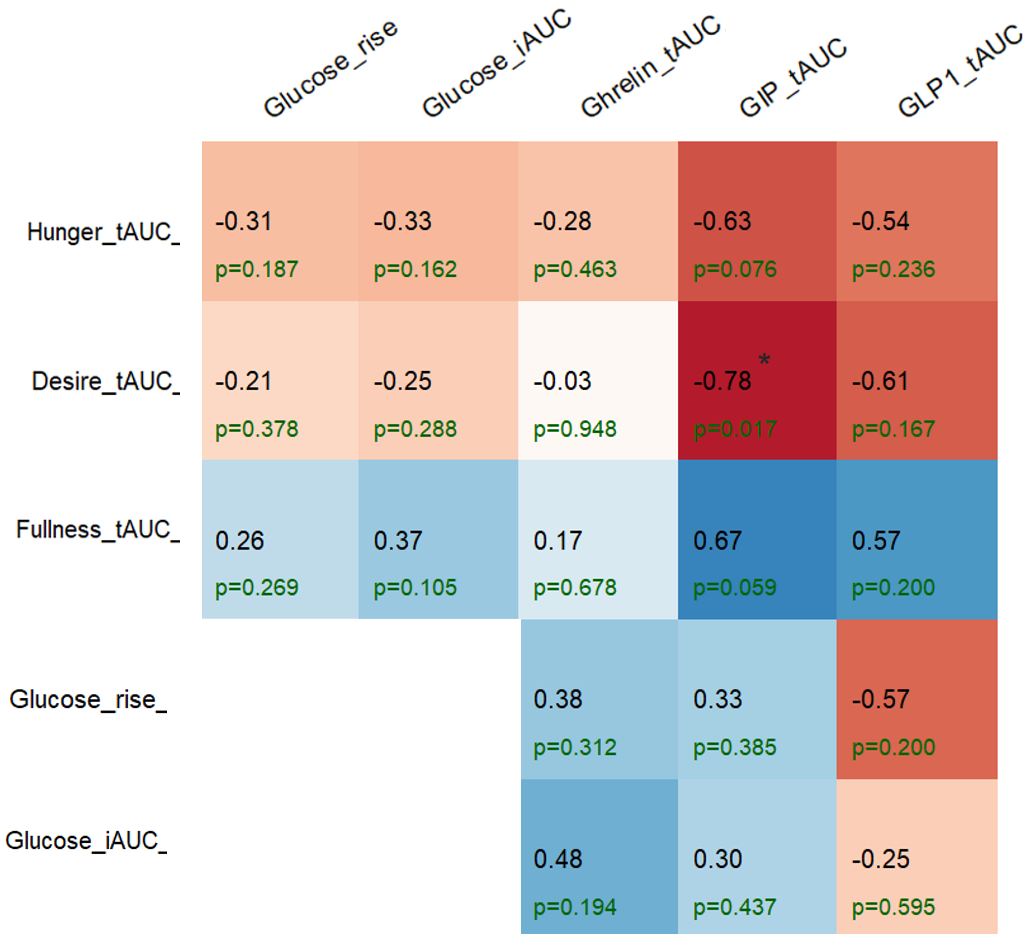


Breakfast 0 – 240min Lunch 240 – 420min


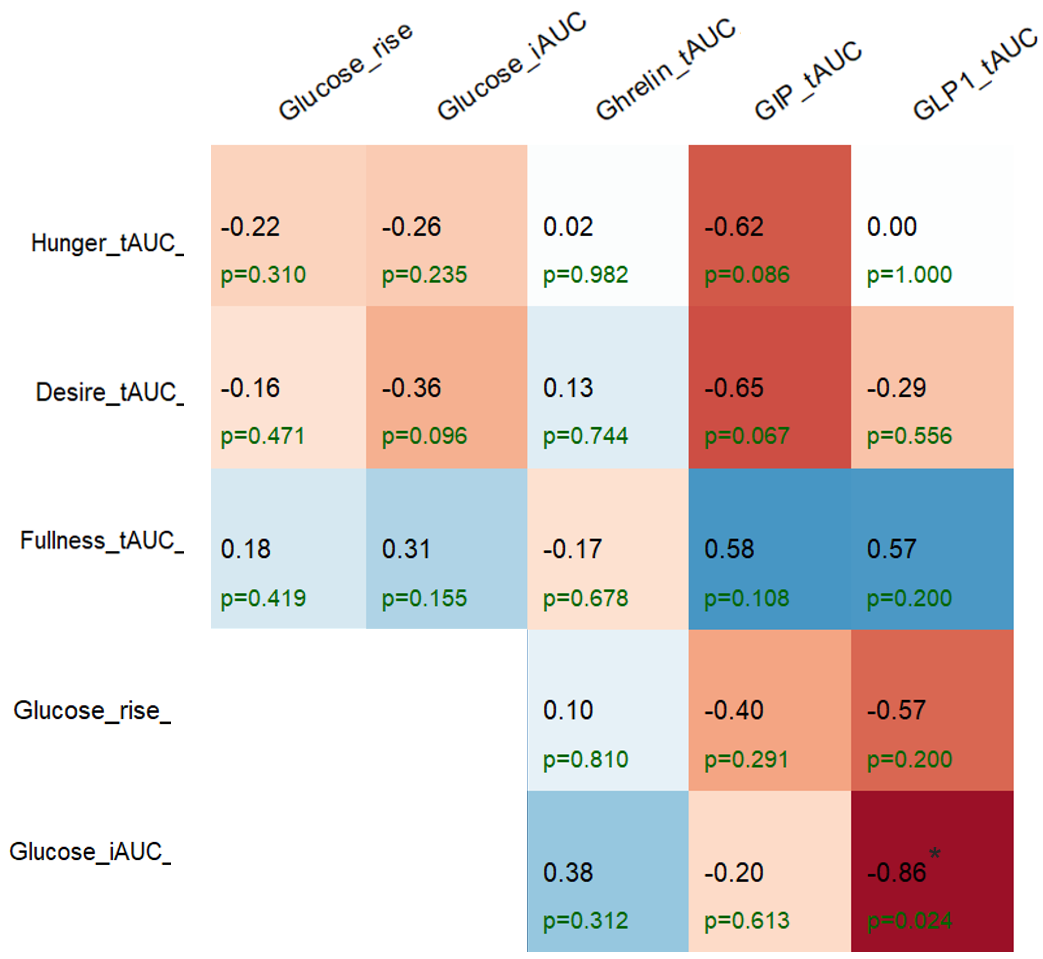

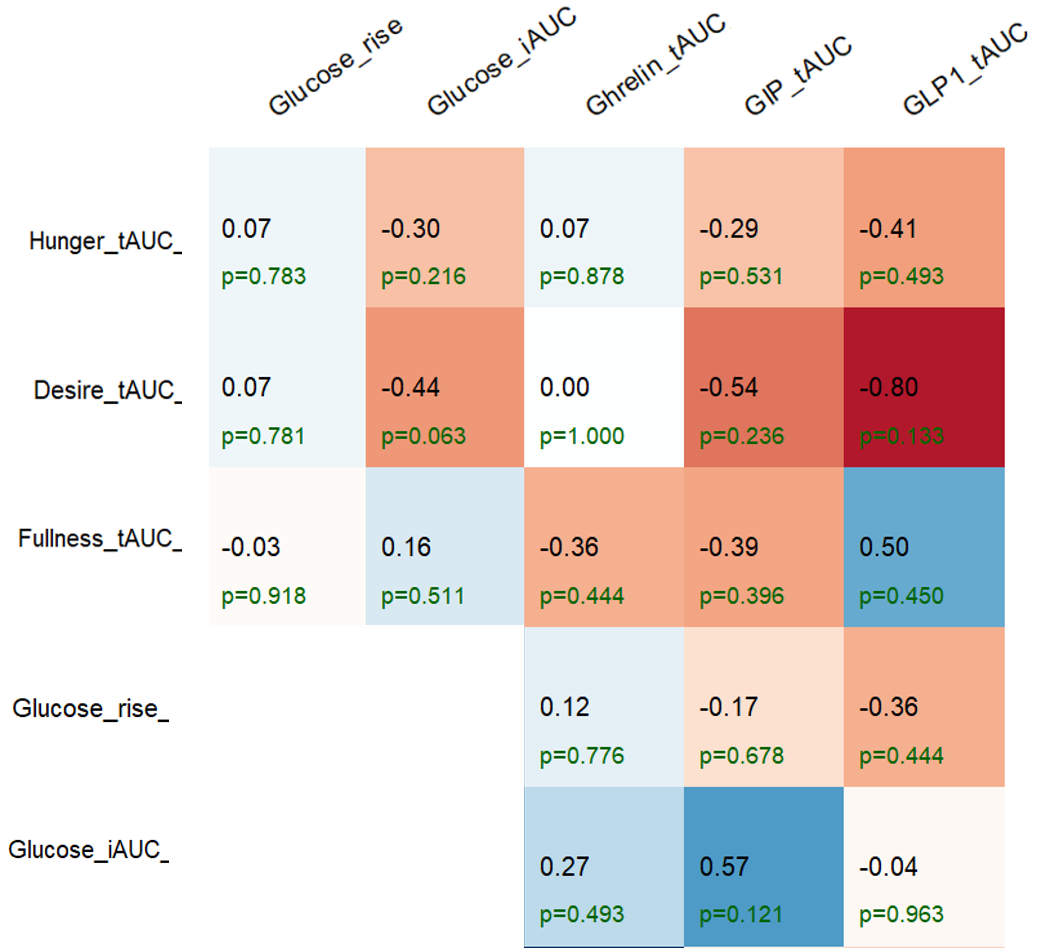


Snack 420 – 660min Dinner 660 – 780min

Spearman rank correlation (r) between postprandial glucose rise, glucose iAUC and hormones: Ghrelin, GIP and GLP-1 measured as tAUC, and subjective appetite measures: fullness, desire to eat and hunger measured as tAUC. Significant correlations are indicated by (p < 0.05) *, *n = 21*.

**Supplementary Table 4.** Percentage increased above fasting concentrations for inflammation markers

| **Inflammation marker** | **Rye** | **Wheat** |
| --- | --- | --- |
| **GlycA** | 10,7% | 6,2% |
| **GlycB** | 11% | 9% |
| **SPC** | 15,1% | 7,6% |

**Supplementary Table 5.** The percentage of participants where inflammation markers increased above fasting levels with at least 10%.

| **Inflammation marker** | **Rye** | **Wheat** |
| --- | --- | --- |
| **GlycA** | 44% | 9% |
| **GlycB** | 44% | 45% |
| **SPC** | 56% | 36% |
